# Application of network pharmacology in the study of the mechanism of action of traditional chinese medicine in the treatment of COVID-19

**DOI:** 10.3389/fphar.2022.926901

**Published:** 2022-08-04

**Authors:** Shihao Zheng, Tianyu Xue, Bin Wang, Haolin Guo, Qiquan Liu

**Affiliations:** ^1^ Graduate School, Hebei University of Traditional Chinese Medicine, Shijiazhuang, China; ^2^ Department of Spleen and Stomach, First Affiliated Hospital of Hebei University of Traditional Chinese Medicine, Shijiazhuang, China

**Keywords:** network pharmacology, COVID-19, mechanism of action, traditional Chinese medicine pharmacology, review

## Abstract

Network pharmacology was rapidly developed based on multidisciplinary holistic analysis of biological systems, which has become a popular tool in traditional Chinese medicine (TCM) research in recent years. Its characteristics of integrity and systematization provide a new approach for the study on complex TCM systems, which has many similarities with the holistic concept of TCM. It has been widely used to explain the mechanism of TCM treatment of diseases, drug repositioning, and interpretation of compatibility of TCM prescriptions, to promote the modernization of TCM. The use of TCM have provided crucial support on prevention and treatment of diseases such as the famous “three medicines and three prescriptions”. Furthermore, TCM has become an important part of the treatment of COVID-19 and is one of the main contents of the “Chinese plan” to fight the epidemic. The current review demonstrated the role of TCM in treating diseases with multiple components, multiple targets, and multiple pathways, interprets the connotation of TCM treatment method selection based on pathogenesis and also discusses the application of network pharmacology in the study of COVID-19 treatment in TCM including single drug and prescription. However, there are still some shortcomings such as the lack of experimental verification and regular upgrading of the TCM pharmacology network. Therefore, we must pay attention to the characteristics of TCM and develop a network pharmacology method suitable for TCM system research when applying network pharmacology to TCM research.

## Introduction

Since December 2019, a novel coronavirus outbreak has occurred in various parts of the world. On 12 March 2020, the World Health Organization declared the novel Coronavirus 2019 (COVID-19) a global pandemic and the world’s sixth public health emergency, which is causing a serious public health threat worldwide. More and more countries were involved, and tens of millions of people around the world are at great risk. It has been confirmed that the infection process caused by SARS-COV-2 is closely related to angiotensin-conversion enzyme 2 (ACE2). After binding the ACE2 receptor, the spike protein of SARS-COV-2 enters the human body and attacks new cells of the body, which promotes the reproduction of the virus and results in serious consequences (Xu, et al., 2020a; Zhou, et al., 2020). At present, COVID-19 patients are often accompanied by complications of multiple organ damage, with the main clinical symptoms including fever, diarrhea, cough, muscle pain, and fatigue (Guan, et al., 2020; Huang, et al., 2020). It has been reported that COVID-19 has the largest non-fragment genome among all RNA virus species, with a length of about 30 kb (Li, et al., 2020). Because of this, the plasticity of the genome is enhanced, which increases the possibility of recombination and mutation of the genome, and improves its chances of cross-species transmission and genetic diversity (Ji, et al., 2020).

Today, COVID-19 poses a serious threat to human health. As of now, there are no specific small molecule antiviral drugs for the treatment of this infectious disease, while effective prevention and treatment are of vital importance worldwide. Some time ago, some western medicine anti-inflammatory drugs and antiviral drugs in patients with symptoms and elimination of the virus have shown a certain effect (Xu, et al., 2020b; Grein et al., 2020; Nili, et al., 2020). And with the development of the Novel Coronavirus vaccine, the world’s attention has shifted from curing disease to preventing disease, and there has been a new level of awareness. In Traditional Chinese medicine, COVID-19 has also been considered an epidemic disease, in which the human body is attacked by toxins and pathogens from outside. The application of TCM adopts the holistic concept, combined with signs, symptoms, and other clinical manifestations, to seek the causes and examine the syndromes, to support the selection of Chinese medicines based on syndrome differentiation (Wang, et al., 2020b). Its disease location is mainly the lung, spleen, and stomach, and its pathogenesis is inseparable from the five kinds “dampness, heat, poison, stasis, and deficiency” (Wang, et al., 2020a; Chen, et al., 2020). Since ancient times, TCM has accumulated valuable experience in the treatment of respiratory infectious diseases, and has embodied unique advantages and characteristics in the prevention and treatment of pneumonia (Zhang, et al., 2020b; Wei, 2020). Based on in-depth research on phytochemical and molecular biological methods as well as screening natural compounds, Zhang D. H. et al. (2020) found that 13 components present in traditional Chinese medicine also have potential anti-2019-nCoV activity, and network pharmacology analysis predicted that the treatment of diseases with these herbs is related to hypoxia response, immune/inflammation reactions, and regulating viral infection. Another study showed that after screening medicinal plants, further pharmacological studies found a variety of medicinal plants can simultaneously regulate host inflammation responses and have potential *in vivo* anti-novel coronavirus effects (Ling, 2020). The early intervention of traditional Chinese medicine effectively alleviated the further deterioration of the disease, while the treatment of integrated traditional Chinese and Western medicine reduced the complications and adverse reactions caused by antibiotics and glucocorticoids (Dai, et al., 2020). Fan et al. (2020) conducted a systematic review and meta-analysis by searching multiple electronic databases and strictly implementing the inclusion criteria, in which seven original studies including 732 adults finally confirmed that TCM, as a treatment with standard care, helps us to improve treatment outcomes in the COVID-19 cases. Fuzimoto (2021) collected a large amount of literature and found through multiple studies that constituents of *Artemisia annua* can impede the viral transcription and replication process, hinder the SARS-CoV-2 attachment, membrane fusion, and internalization into the host cells. The constituents also can modulate the host immune response to fight the infection.

Based on the previous innovation of “one drug, one target, one disease”, which resulted in a high clinical failure rate of new drugs, Hopkins proposed the concept of network pharmacology in 2007 by constructing a biological network of “drug-component-target-disease” to analyze the mechanism of drugs affecting diseases (Hopkins, 2007; Hopkins, 2008). Network pharmacology is an emerging discipline based on bioinformatics, high-throughput omics data analysis, computer technology, online database, and other technologies to explore the occurrence, development and treatment process of diseases from the perspective of biological network. The new method can further study the mechanism of drug treatment of diseases and guide the development of new drugs (Li and Zhang, 2013; Wu, et al., 2016; Zhang, et al., 2019) which helps us to have a more comprehensive understanding of the therapeutic effect of drugs and the basis of disease. It provides a better reference for the treatment of diseases and the invention of clinical new drugs. The network theory of “drug-target-gene-disease” in network pharmacology and its holistic analysis of biological systems coincide with the holistic concept proposed by traditional Chinese medicine. Network pharmacology is also characterized by systematization. It can reposition a single target or multiple targets in the entire biological network, accelerate the discovery of drug targets, and contribute to the research and development of new drugs. Moreover, network pharmacology can analyze the adverse effects of drugs and ensure the safety of drugs. Network pharmacology has been widely accepted and applied in Chinese medicine since it was put forward. Its unique advantages provide new ideas and references for the study of the complex Chinese medicine system. This paper reviews the current research on the mechanism of TCM in the treatment of COVID-19 based on network pharmacology, in order to find the deficiencies in relevant research fields, broaden the ideas for future research, and make a contribution to the development of human medicine.

## Application of network pharmacology in the field of traditional Chinese medicine

### Network pharmacology research techniques

In recent years, the development of network pharmacology is inseparable from the innovation and development of many research technologies, including network visualization technology, omics technology, high-throughput technology, network analysis technology, molecular interaction technology, computer technology, etc. The application of these technologies is conducive to improving the accuracy of prediction models in network pharmacology research. And can help us to improve the construction of biological networks, network pharmacology omics technologies including metabolomics, proteomics, and genomics, etc., can be more effective to observe drugs at different levels play a role of regulation and control, network visualization technology through tools such as guess, reflect the whole process of the network will appear more image, intuitive, Network analysis technology can carry out technical analysis on the established network, obtain useful information more effectively and improve efficiency, the complex connections and huge information in the biological network are transformed into a more intuitive and recognizable visual network, which effectively reduces the difficulty of text information and facilitates further research. Molecular interaction technologies mainly include nano-liquid chromatography-mass spectrometry (Hsieh and Korfmacher, 2006), biofilm interference (Wartchow, et al., 2011), and plasma resonance (Guo, 2012), which can directly reflect the interaction between proteins and drug molecules. High-throughput technology can detect a large number of detection indicators at a time on the premise that the tissues and cells are intact, with good real-time performance and the advantages of visualization. Computational prediction is the bridge between experiment and theory, and the rational application of computer technology can help to narrow down the large libraries of compounds into a subset with limited resources in a short time and provide theoretical guidance for future clinical drug use (Ling, 2020). Yang et al. combined computer science with network pharmacology, through transcriptomic analysis of gene expression after Ma Xing Shi Gan Decoction (MXSG) administration in a rat model of LPS-induced pneumonia, the Toll-like receptor (TLR) and thrombin signaling pathway were proposed to be vital pathways for MXSG mediated anti-inflammatory effects (Yang, et al., 2020). Although these network pharmacology research technologies have made some achievements and brought us great convenience, they are still in the initial stage and still need further research and exploration.

### Network pharmacology application and traditional Chinese medicine research

#### To expound the mechanism of TCM in treating diseases

By integrating various types of data and using network analysis tools such as network analysis and target prediction, the key active ingredients that may play a role in the treatment of diseases in Traditional Chinese medicine are accurately screened by using the network pharmacology analysis method, and their specific mechanism of action is clarified so as to promote the discovery of new drugs. This kind of research is generally based on the drug-component-target-disease biological network and TCM component database constructed by relevant information among drugs, targets, and diseases, and excavates the key nodes in the biological network to further clarify the possible mechanism of TCM action. It will provide new ideas and references for solving the problems of unclear specific effective components and unclear mechanisms in treating diseases in the future.

### Reorientation of drugs

Treating diseases according to the pathogenesis of diseases is a unique characteristic of the TCM treatment system, which coincides with the connotation of precision treatment in modern medicine. Network pharmacology Through the construction of the biological network of “drug-component-target-pathway”, while obtaining the key components, core targets, and related signal pathways of traditional Chinese medicine, possible new indications of traditional Chinese medicine can be speculated based on different signal pathways to further analyze the pharmacological mechanism of traditional Chinese medicine ingredients. The main research strategies include drug-target topological similarity, ligand-based similarity, ligand-receptor reverse docking, etc. Reduning injection can treat influenza in clinical practice, and some studies have found that the effect on cardiovascular diseases, tuberculosis, diabetes and other clinical treatment is also very significant (Luo, et al., 2015).

### Interprets the compatibility of Chinese medicine prescriptions

The effect of Chinese medicine with compatibility to treat disease is accurate and precise, and the emerging regularity of network pharmacology for science provides a new method based on traditional Chinese medicine composition and the interaction of multiple targets for network pharmacology research. The analysis of the effect of drugs on the disease target network further reveal the compatibility of Chinese characteristics. According to the number of active ingredients and targets of each traditional Chinese medicine to determine the importance of the position in the prescription can better promote the modernization of traditional Chinese medicine development. Some scholars conducted an in-depth study on the key active ingredients and component relationship of Yujin Prescription in the treatment of cardiovascular and cerebrovascular diseases and found that turmeric plays the most important role in the treatment of diseases. Musk, gardenia, and borneol play an assisting role in increasing the curative effect of turmeric prescription (Tao, et al., 2013).

## The application of network pharmacology in the study of TCM treatment of COVID-19

In clinical treatment of COVID-19, TCM also follows the holistic concept and the principle of treating diseases according to pathogenesis. However, few scholars have been able to comprehensively and systematically explain the specific mechanism of TCM treating diseases. From ancient times up to now, the holistic concept has always been an important idea in the treatment of diseases in Traditional Chinese medicine. Pharmacology, an emerging discipline network, also starts from the holistic perspective and deeply interprets the mechanism of drugs in the treatment of diseases by constructing a biological network of “drug-component-target-pathway”. At present, a large number of studies have been carried out on the application of network pharmacology to explore the mechanism of traditional Chinese medicine on COVID-19. Based on the above application of network pharmacology in the study of traditional Chinese medicine for the treatment of COVID-19, it is summarized as follows: Single drug study and prescription study of Traditional Chinese medicine (Table 1). The main signal pathways involved are introduced as follows (Table 2).

**TABLE 1:** Network pharmacology analysis of TCM in the treatment of COVID-19.

References	Formulae & Herbs	Objects	Main ingredients	Main targets	Main signaling pathways	Outcome
Wang et al. (2021a)	R. Crenulata	Molecular Docking	Quercetin, Kaempferol, Tamarixetin	IL6, IL1B, TNF	TNF, IL-17	R. crenulata can play an immunoregulatory and anti-inflammatory role in the cytokine storm of COVID-19
Yu et al. (2021)	Banlangen	None	Acacetin, Isovitexin, Isaindigodione	PTGS1, PTGS2, PPARG	TNF, IL-17, RAGE	Banlangen has a potential pharmacological effect on the treatment of COVID-19
Mu et al. (2021)	Rhizoma Polygonati	Molecular Docking	Diosgenin, Beta-sitosterol, Sitosterol	CASP3, TP53, PTGS2	MAPK, Hepatitis B, Pathways in cancer	Potential compounds in Rhizoma Polygonati can act on different targets with cancer and viral related signaling and have a potential in treatment of COVID-19
Qin et al. (2021)	Pueraria root	Molecular Docking	Puerarin	TNF, CASP3, CASP8	IL-17, MAPK, Apoptosis	Related target genes of puerarin can regulate lung inflammation and oxidative stress response
Banerjee et al. (2021)	AP	None	Andrographolide	FABP5, FGF1, GLI1	MAPK, Toll-like receptor, PI3K/AKT	Andrographolide can reduce the production of pro-inflammatory factors and cytokines in viral infection
Xiao et al. (2021)	Resverarum	Molecular Docking	Resveratrol	MMP9, MMP13, KCNH2	TNF, NF-κB, IL-17	Resveratrol can further alleviate the hyperinflammatory response of COVID-19
Liu et al. (2021)	Sophora flavescens Ait	Male C57BL/6 mice	Matrine	TP53, AKT1, IL6	C-type lectin receptor, PI3K-Akt, Toll-like receptor	Matrine may achieve simultaneous intervention of COVID-19 combined with liver injury by multi-dimensional pharmacological mechanism
Gao et al. (2020)	Ephedra-bitter almond	Molecular Docking	β-sitosterol, Stigmasterol, Estrone	PTGS2, HSP90AA1, AR	IL-17, TNF, PI3K-Akt	Ephedra-bitter can treat and prevent COVID-19 by inhibiting virus reproduction, regulating immune response and promoting body repair
Li et al. (2021b)	Ephedra-Glycyrrhiza pair	Molecular Docking	DehydroglyaspErins C, Phaseol, Gancaonin H	FOS, PTGS2, IL2	PI3K-Akt, JAK-STAT	Ephedra-Glycyrrhiza pair can control PI3K-Akt signaling pathway to exert organ protection, antiviral and immune regulation effects
Lin et al. (2021)	Yinqiao powder	Molecular Docking, SPR assay	Quercetin, Naringin, Luteolin	TP53, MAPK3, IL-6	MAPK, TNF, Toll-like receptor, T-cell receptor	Its antagonistic action against COVID-19 is mainly related to the regulation of inflammation-related proteins
Li et al. (2021a)	SHXP	Molecular Docking	Quercetin, Kaempferol, Luteolin	IL6, IL10, EGFR	HIF-1, NF-κB, Toll-like receptor	SHXP is a treatment for severe COVID-19 by regulating the cytokine storm of the human immune system and inhibiting viral growth
Tao et al. (2020)	HSBDF	Molecular Docking	Baicalin, Quercetin	MAPK3, MAPK8, TP53	TNF, MAPK, PI3K-Akt, Nod-like receptor	Quercetin and baicalein in HSBDF can regulate multiple signaling pathways through ACE2, which may play a therapeutic role on COVID-19
Li et al. (2021c)	MXSGD	IL-6 induced rat lung epithelial type II cells	Amygdalin, Spinacen, Euchrenone	STAT3, BAX, CASP3	JAK-STAT	The key target of MXSGD can effectively inhibit the damage of RLE-6TN cells and exert its therapeutic effects through the JAK-STAT signaling pathway
Wang et al. (2021b)	MXYGD	Molecular Docking	Quercetin, Luteolin, Formononetin	IL4, IL6, CASP3	PI3K-Akt, Chemokine, HIF-1	MXYGD plays an significant role in the prevention and treatment of COVID-19 by activating lymphocytes, T cells,leukocytes, and other immune and anti-inflammatory effects
Xia et al. (2020)	LQC	Molecular Docking, AKT1 encoding protein	Beta-carotene, Kaempferol, Luteolin	AKT1, JUN, MAPK8	Toll-like receptor, IL-17, TNF	LQC may provide a potentially effective treatment for COVID-19, reducing lung tissue damage and effectively relieving symptoms
Zheng et al. (2020a)	LHQW	None	Kaemperol, Luteolin, Catechin	PIK3CG, TLR3, PRKCB	JAK-STAT, Pathways in cancer	LHQW modulates the inflammatory process, repairs lung injury and exerts antiviral effects
Kong et al. (2020)	PDL	Molecular Docking	Oxysophocarpine, Isoacolamone, Adenosine	EGFR, TNF, IL-6	Toll-like receptor, MAPK	PDL may have therapeutic effects on COVID-19 through three aspects, including the anti-virus entry into cells,moderate immune system, and anti-inflammation
Yan et al. (2020)	Qingfei Paidu Decoction	Molecular Docking	Coniferin, Gallic, Delphinidin	AGTR1, TNF, CASP3	Apoptosis, RAS	Qingfei Paidu decoction might treat COVID-19 through its multiple medicinal ingredients that have various targets and signaling pathways
Ye et al. (2021b)	SQW	Molecular Docking	Luteolin, Quercetin, Kaempferol	ACE2, IL6, NOS3	HIF-1, TNF, IL-17	SQW can have anti-COVID-19 effects through the immune-inflammatory pathway
Zheng et al. (2020b)	XBJ	Molecular Docking	5-hydroxymethyl-furfural, Albiflorin, Apigenin	ALB, EGFR, TNF	HIF-1, TNF, PI3K-Akt, NF-κB	XBJ can regulate different genes, act on different pathways, and synergize immunoregulatory and anti-inflammatory effects in COVID-19
Xing et al. (2020)	XBJ	Molecular Docking	Luteolin, Apigenin, Quercetin	IL6, MAPK1, TNF	Toll-like receptor, TNF, T cell receptor, Prolactin	XBJ could play the role of anti-inflammatory, immune responseand anti-virus to treat COVID-19
Ye et al. (2021a)	TGQ	Molecular Docking, SPR experiment	Ritonavir, Ribavirin, Lopinavir	IL6, PTGS2, TNF	IL17, TNF	TQG might have a therapeutic effect on COVID-19 by regulating immune, inflammation and viral infection related targets and pathways
Han et al. (2020)	CDPF	Molecular Docking	Quercetin, Luteolin, l-tyrosine	IL6, TNF, IL10	TNF, Oxytocin, MAPK	CDPF mainly plays antiviral, immunomodulatory and anti-inflammatory effects in the treatment of COVID-19
Xu et al. (2021)	Chansu injection	Molecular Docking	Bufalin, Resibufogenin, Cinobufagin	AKT2, GRB2, EGFR	JAK-STAT, PI3K-Akt, MAPK, FoxO	Chansu injection could regulate activate immune cells, virus duplication, and alleviate inflammatory responses through the JAK-STAT and PI3K-Akt signaling pathways
Zhang et al. (2020c)	RDS	Molecular Docking, Phytochemical analysis of RDS	Luteolin, Quercetin, Fisetin	NCOA1, RXRA, NCOA2	Prolactin, IL17, HIF-1	RDS has certain preventive and therapeutic effects on early COVID-19

**TABLE 2:** Key signaling pathways in the treatment of COVID-19.

No.	Name	Entry	Class	Disease	References
1	Pathways in cancer	map05200	Human diseases, Cancer: overview	COVID-19	Mu et al. (2021)
					Zheng et al. (2020a)
2	NF-κB	map04064	Environmental information processing Signal transduction	COVID-19	Xiao et al. (2021)
					Li et al. (2021a)
					Zheng et al. (2020b)
3	IL17	map04657	Organismal systems, Immune system	COVID-19	Wang et al. (2021a)
					Yu et al. (2021)
					Xia et al. (2020)
4	MAPK	map04010	Environmental information processing, Signal transduction	COVID-19	Banerjee et al. (2021)
					Lin et al. (2021)
					Han et al. (2020)
5	PI3K-Akt	map04151	Environmental information processing, Signal transduction	COVID-19	Li et al. (2021b)
					Tao et al. (2020)
					Xu et al. (2021)
6	TNF	map04668	Environmental information processing, Signal transduction	COVID-19	Ye et al. (2021a)
					Xiao et al. (2021)
					Gao et al. (2020)
7	JAK/STAT	map04630	Environmental information processing, Signal transduction	COVID-19	Li et al. (2021b)
					Li et al. (2021c)
					Xu et al. (2021)
8	HIF-1	map04066	Environmental information processing, Signal transduction	COVID-19	Wang et al. (2021b)
					Ye et al. (2021b)
					Zhang et al. (2020c)
9	Toll-like receptor	map04620	Organismal systems, Immune system	COVID-19	Liu et al. (2021)
					Kong et al. (2020)
					Xing et al. (2020)
10	Apoptosis	map04210	Cellular processes, Cell growth and death	COVID-19	Qin et al. (2021)
					Yan et al. (2020)

### Single drug study of traditional Chinese medicine

Some studies have applied network pharmacology to explore the treatment of COVID-19 with Tibetan Hongjingtian and found that four active ingredients play a significant role, such as quercetin and kaempferol, whose related target genes act on the immune response and inflammatory response, especially the TNF signaling pathway and IL-17 signaling pathway, the molecular docking also showed that the core compound quercetin had a considerable degree of affinity with IL-6, IL-1β, and TNF-α (Wang, et al., 2021a). In the research on the molecular mechanism of the therapeutic effect of *Radix Isatidis* (Banlangen) on COVID-19, 33 active ingredients were screened out by searching the database, and 11 co-targets with the disease were identified. Through tetrapyrrole binding, protein phosphatase binding, and the apoptotic process involving cysteine-type endopeptidase. Biological processes such as activity play a therapeutic role in TNF signaling pathway and IL-17 signaling pathway. Ultimately, Banlangen inhibits immune response and inflammation by acting on NOS2, PTGS1, PTGS2 and other targets, thereby efficiently resists COVID-19 (Yu, et al., 2021). *Rhizoma Polygonati*, a herb with a homology to medicine and food, has become one of the key TCM treatments for COVID-19 during the current epidemic. Studies have identified 10 active components that may have therapeutic effects on COVID-19 in *Rhizoma Polygonati* and screened out 23 possible targets and 179 metabolic pathways. It mainly involves MAPK signaling pathway, Hepatitis B metabolic pathway, Pathways in cancer, etc., and plays a therapeutic role through the regulation of inflammatory response, DNA damage repair, apoptosis, redox, and cell metabolism (Mu, et al., 2021). Additionally, *Rhizoma Polygonati* has been shown to effectively enhance the immune response when treating lung inflammation in mice, thereby eliminating the CYTOKine storm of COVID-19 and playing an anti-inflammatory role (Zhao, et al., 2018). Puerarin, as one of the important components of *Pueraria* root, has an important regulatory mechanism for COVID-19, and its pharmacological function cannot be ignored. Qin et al. found that the key targets of Puerarin in the treatment of COVID-19 include TNF, CASP3, CASP8, IL2, EGFR, PRKCB, and PRKCA, NOS3, PPARG, TP53, etc. These target genes regulate oxidative stress response and lung inflammation by acting on the IL17 signaling pathway, MAPK signaling pathway, apoptosis pathway, and TNF signaling pathway (Qin, et al., 2021).

Other studies have found that andrographolide, the main component of *Andrographis Paniculata*, has significant effects on upper respiratory tract infection and viral infection, and can significantly reduce the production of pro-inflammatory factors and cytokines in viral infection. By acting on the MAPK signaling pathway, Toll-like receptor pathway, PI3K/AKT signaling pathway, etc., it plays an antiviral role and provides immune protection to the body, thus ultimately achieving the effect of COVID-19 treatment (Banerjee, et al., 2021). Resveratrol, found in the Chinese herb *Resverarum*, has previously been shown to have anti-inflammatory and antiviral effects and is now a candidate for COVID-19 treatment. Xiao’s study found that the core targets of Resveratrol in the treatment of COVID-19 mainly reduced the expression level of cytokines by acting on the TNF signaling pathway, NF- Kappa B signaling pathway, and IL-17 signaling pathway, thus further alleviated the hyperinflammatory response of COVID-19. The molecular docking results showed that Resveratrol could form a strong interaction with MMP13 and PLAT (Xiao, et al., 2021). Furthermore, some scholars found that resveratrol can not only reduce the respiratory syncytial virus-induced in the mouse model of interferon -γ, but also can reduce the titer of the respiratory syncytial virus as well as reduce airway inflammation and high responsiveness of the body, of which the effect is significant (Zang, et al., 2011; Zang, et al., 2015).

In addition, there are also clinical studies on TCM for the treatment of COVID-19, which also play an important role in interpreting the mechanism of TCM for the treatment of COVID-19. Liu et al. found that the potential therapeutic mechanism of matrine on COVID-19 combined with liver injury is closely related to improving the immune system, regulating the level of inflammatory factors, and regulating the antiviral process. It can significantly reduce the expression of TP53, AKT1, IL6, and TNF in mice, while Real-time RT-PCR also demonstrated that matrine can reduce the expression of TP53, AKT1, IL6, TNF and ATM in mice with lung injury or liver injury (Liu, et al., 2021). *Ephedra-Bitter* is a common drug pair in the prescription for the treatment of COVID-19. Clinical practice has proved that it has a good therapeutic effect on pulmonary inflammatory diseases. Gao et al. verified through network pharmacological analysis that β -sitosterol, stigmasterol, and estrone, the key components of *Ephedra-Bitter*, had a high binding activity with ACE2 and 3CL, providing a new molecular structure for the development of novel Coronavirus new drug. *Ephedra-Bitter* can prevent and treat COVID-19 by regulating immune response, inhibiting virus reproduction, and promoting body repair, reflecting the important role of TCM in epidemic prevention and control (Gao, et al., 2020). Nowadays, *Ephedra* and *Glycyrrhiza* are frequently used in the treatment of COVID-19. Some scholars found that Pathway analysis between COVID-19 and *Ephedra-Glycyrrhiza* drug pair showed that FOS, PTGS2, IL2, ALB, and TNF-α were the key targets, and they can control the PI3K-Akt signaling pathway to exert antiviral, organ protection and immune regulation effects (Li, et al., 2021b).

### Study on Chinese medicine prescription

TCM prescriptions are more effective in the treatment of COVID-19, and their mechanism of action is more complex, which is worth further study. Some studies have confirmed that Yinqiao Powder contains a total of 30 important active ingredients after the screening, but only quercetin, naringin, and luteolin have been quality verified. Its central proteins are TP53, MAPK3, IL-6, TNF, etc. By acting on the MAPK signaling pathway, TNF signaling pathway, Toll-like receptor signaling Pathway, T-cell receptor signaling Pathway, etc., play a role in the treatment of COVID-19, its antagonistic action against diseases is mainly related to the regulation of inflammation-related proteins, and the study also confirmed that ACE2 and luteolin have the best binding ability, mainly through π–π bonds or hydrogen bonds, which means that luteolin has certain effects in preventing COVID-19 infection (Lin, et al., 2021). Earlier, scholars found that MAPK3 protein is closely related to the inflammatory response of lung injury. By inhibiting MAPK1/MAPK3, the levels of pro-inflammatory factors such as IL-1β and TNF-α can be effectively reduced, thus playing an anti-inflammatory role and eliminating lung inflammation (Di Paola, et al., 2009). Network pharmacological analysis of Suhexiang Pill (SHXP) found that 326 compounds were screened out, such as quercetin and so on, among which 6 central genes were closely related to the treatment of diseases, including IL6, IL10, EGFR, TNF, etc. Through GO enrichment analysis, its therapeutic effects are mediated by synergistic regulation of multiple biological signaling pathways, including Interleukin 17, Toll-like receptor, Nuclear factor kappa-b, Janus kinase/STAT3and hypoxia-inducible factor 1 signaling Pathways SHXP works to treat severe COVID-19 by moderating cytokine storms inhibiting viral growth and regulating the body’s immune system (Li, et al., 2021a). Studies have proved that TNF signaling pathway is an important pathway of the inflammatory response, and its related factor receptors can play a role by inducing apoptosis, while quercetin, an important component, also plays an anti-inflammatory role by regulating TNF signaling pathway (Berntsen, et al., 2018; Nishikawa, et al., 2018; Pang, et al., 2018). Other studies confirmed that Huashi Baidu Formula (HSBDF) compound-target network for COVID-19 treatment contains 178 related compounds and 272 corresponding targets, it mainly acts on TNF signaling pathway, MAPK signaling pathway, Nod-like receptor signaling Pathway, PI3K-Akt signaling Pathway, etc., while baicalin and quercetin are the two compounds with the strongest affinity with ACE2 in HSBDF. By regulating the above pathways to play a role in the treatment of COVID-19, also quercetin and baicalein were both flavonoids, they can reduce the barrier dysfunction induced by influenza A virus by inhibiting the NOX4/NF-jB/MLCK pathway (Tao, et al., 2020). It has long been confirmed that baicalin can play an anti-pulmonary fibrosis role through the PI3K-Akt signaling pathway (Duronio, 2008; Zhao, et al., 2012; Wang, et al., 2018).

Maxing Shigan decoction (MXSGD) is a common prescription for treating exogenous diseases. In the study exploring its treatment of COVID-19, it was found that 105 key active ingredients involved 1,025 possible targets were screened out, these targets can effectively inhibit the effective expression level of il-6-mediated jak-Stat signaling pathway related proteins, improve the expression level of Bcl-2 protein, reduce the expression level of Caspase 3, Bax, P-JAK2 and other proteins, so as to effectively inhibit the damage of RLE-6TN cells and exert its therapeutic effect, it reflects the characteristics of multi-component, multi-target and multi-pathway intervention for COVID-19 (Li, et al., 2021c). Different from this, some scholars have found that Maxingyigan Decoction (MXYGD) for the treatment of COVID-19 contains three key components quercetin, luteolin, and formononetin, and also has three pivotal target genes, namely IL4, IL6, and CASP3, the results of molecular docking validation showed that the IL6 receptor and quercetin had the highest docking score, and the other three key components scored higher than Ribavirin. MXYGD plays an important role in the treatment and prevention of COVID-19 by activating leukocytes, T cells, lymphocytes, and other immune and anti-inflammatory effects (Wang, et al., 2021b). Today, Lianhua Qingwen Capsule has achieved good efficacy in the clinical treatment of COVID-19, which is also recognized by many scholars around the world (Zheng, et al., 2020a). Through network pharmacology analysis, it was found that Lianhua Qingwen Capsule can play a role by regulating apoptosis, viral infection and immune response, and molecular docking of Akt1 gene, the most important gene of Lianhua Qingwen Capsule, was found to be involved in pulmonary fibrosis, viral infection and lung injury. Thus providing a potential and effective treatment for COVID-19, reducing lung tissue damage, helping to eliminate the viral infection in the lungs, and effectively relieving symptoms (Xia, et al., 2020). Through network pharmacology analysis, Zheng S. et al. (2020) found that Lian Hua Qing Wen (LHQW) can exert antiviral effects, repair lung injury, and modulate the inflammatory process, and it also improves ACE2-expression-disorder-caused symptoms and relieves the “cytokine storm”. Pudilan (PDL) has also been reported to have the potential to treat COVID-19, but its underlying mechanisms are still not fully understood. Kong et al. selected 68 targets of PDL for the treatment of COVID-19 by network pharmacology method and found that PDL could block SARS-COV-2 from entering cells by blocking ANGIOtensin-converting enzyme 2 through GO enrichment analysis and KEGG signaling pathway enrichment analysis. By affecting EGFR, TNF, IL-6, and other proteins to inhibit cytokine storm, the regulation of COVID-19 include anti-inflammatory, antiviral, and immunomodulatory effects (Kong, et al., 2020).

Qingfei Paidu formulas is one of the key formulae developed in China to treat COVID-19, which has been widely recognized by scholars around the world. Studies have confirmed that it has a good clinical effect on the treatment of confirmed cases. Yan et al. found that Qingfei Paidu formulas contain 18 kinds of Traditional Chinese medicines, including 163 key active ingredients, which play a therapeutic role in clinical treatment by regulating AGTR1, TNF, CASP3, DPP4, ACE, and other 10 proteins to activate apoptosis signaling pathways. This treatment also has the characteristics of multi-drug, multi-component, multi-target, and multi-pathway, which provides a reference for the mechanism and pharmacological action of Qingfei Paidu formulas to treat COVID-19 in the future (Yan, et al., 2020). As one of the traditional prescriptions, Shenqi Wan (SQW) also has a certain therapeutic effect on COVID-19. Ye X. W. et al. (2021) found that SQW could play a crucial role in the treatment of COVID-19 by directly or indirectly combining luteolin, quercetin, and kaempferol with 3CLpro, ACE2, PTGS2, and PLpro to regulate signaling pathways and multiple biological functions, molecular docking results also showed that the key components of SQW such as kaempferol, quercetin, luteolin and other COVID-19-related targets such as 3CLpro, ACE2, PTGS2, PLpro have the good binding ability. Some researchers found that Chinese medicine Xuebijing (XBJ) played a positive role in the treatment of mild coronavirus disease 2019 (COVID-19) cases. By constructing the “drug-ingredient-target-pathway” effector network, the researchers screened out 14 key targets of XBJ in the treatment of diseases, such as ALB, EGFR, TNF, MAPK1, STAT3, etc. These key targets act on the different signaling pathways and ultimately synergize to produce anti-inflammatory and immunomodulatory effects (Zheng, et al., 2020b). Through another study of Xuebijing (XBJ) injection, Xing et al. found that luteolin, apigenin, quercetin, and other compounds in XBJ injection could affect IL6, MAPK1, TNF and other overlapping targets, which ultimately play the role of anti-virus, anti-inflammatory and immune response to treat COVID-19 (Xing, et al., 2020).

Toujie Quwen Granules (TGQ) mainly have the effect of detoxification and antipyretic, network pharmacology studies have confirmed that TGQ acts on the IL17 signaling pathway and TNF signaling pathway through key targets such as IL6, PTGS2, and TNF, and ultimately controls the infection of the virus and relieves the symptoms of patients (Ye, et al., 2021a). Han et al. (2020) found that the Cold‒Damp Plague Formula (CDPF) could play pharmacological roles against COVID-19 through “multi components‒multi targets‒multi pathways”, mainly involving immune-regulatory, anti-viral, and anti-inflammatory actions. Through the study of Chansu Injection, Xu et al. found that in the treatment of COVID-19, it mainly acts on the PI3K and JAK-STAT signaling pathways through 16 core targets, such as AKT2, GRB2, EGFR, CCNA2, and so on (Xu, et al., 2021). Lung-toxin Dispelling Formula No. 1, referred to as Respiratory Detox Shot (RDS), based on the traditional formula, has played dual effects of pathogen-eliminating and health-strengthening in the treatment of COVID-19. Zhang Z. J. et al. (2020) found through network pharmacology analysis that RDS mainly acts on 42 core targets in the treatment of diseases, involving 134 key components and 29 important pathways, this results in general therapeutic effects for early COVID-19. The above studies have proved that the mechanism of TCM in treating COVID-19 or alleviating adverse symptoms is scientific to a considerable extent, and the application of network pharmacology can better demonstrate the characteristics of multi-components, multi-targets, and multi-pathways of TCM in treating diseases, providing a guarantee for the future research and development of anti-inflammatory drugs.

## Discussion

The development and application of network pharmacology have opened a new idea for studying the mechanism of TCM in treating COVID-19, including all factors related to disease treatment, such as drugs, components, targets, and pathways, which are completely different from the previous linear model of “one drug—one component—one target—one disease”. Network pharmacology technology has significant advantages in the field of Traditional Chinese medicine research on how to discover the basis of pharmacodynamic substances, develop new indications, confirm the mechanism of drug action and study the compatibility of traditional Chinese medicine, etc. The thinking mode is more consistent with the overall concept of treating diseases combining with Traditional Chinese medicine, which provides a reference for the treatment of the current global pandemic COVID-19. In the process of treating COVID-19 and preventing COVID-19 transmission, Chinese physicians have accumulated rich experiences and created three Chinese patent medicines (i.e., Jinhua Qinggan Granules, Lianhua Qingwen Capsule, and Xuebijing Injection) and three herbal prescriptions (i.e., Qingfei Paidu Decoction, Huashi Paidu Recipe, and Xuanfeibaidu Recipe). Furthermore, TCM treatment plays a significant role in reducing the overall mortality rate, shortening the duration of hospitalization, improving the clinical symptoms, preventing disease progression, obtaining favorable treatment outcomes in patients with severe COVID-19, reducing the positive rate of viral nucleic acid testing and improving quality of life and immunity (Xing and Liu, 2021). When network pharmacology is applied to study the mechanism of Traditional Chinese medicine on COVID-19, the multi-component, multi-target and multi-pathway treatment characteristics of network pharmacology can be fully reflected, whether it is single drug or prescription of Traditional Chinese medicine. In these studies, “star targets” or “star pathways” with high frequency were not found, such as TNF and PI3K/AKT signaling pathway, suggesting that Traditional Chinese medicine may regulate pulmonary inflammatory response and immune response, and achieve a comprehensive therapeutic effect on COVID-19. The application of network pharmacology in the study of TCM not only broadens the application scope of single medicine or prescription but also promotes the modernization of TCM, which accords with the development trend of TCM in the future.

Compared with Western medicine, traditional Chinese medicine has significant advantages in relieving symptoms of patients, but its main mechanism of action is not fully understood and needs to be further discovered. Nowadays, due to the influence of many factors, there are still many problems and great challenges in the application of network pharmacology, and most of the results can not be verified or be directly applied to clinical practice. More importantly, the network of the pharmacology of TCM cannot be upgraded regularly, which will cause some errors and incompleteness for us to study the mechanism of traditional Chinese medicine by using this method. Whether we can accurately and comprehensively retrieve the current known information has become the key to applying this method. It is undeniable that the method of network pharmacology with traditional Chinese medicine research appears behind the biological information, the latest scientific research achievements in modern pharmacology and so on. But only depending on its own ingenuity will result in a considerable lag and the magnificent great discovery of artemisinin won't appear again. Therefore, while applying network pharmacology to analyze TCM in the future, we still need to make efforts on exploring more suitable methods for TCM research and contributing to the progress of global medical cause.
